# Growth of centimeter-scale perovskite single-crystalline thin film via surface engineering

**DOI:** 10.1186/s40580-020-00236-5

**Published:** 2020-07-20

**Authors:** Yu-Hao Deng, Zhen-Qian Yang, Ren-Min Ma

**Affiliations:** 1 State Key Lab for Mesoscopic Physics and School of Physics, Peking University, Beijing, China; 2 Frontiers Science Center for Nano-optoelectronics & Collaborative Innovation Center of Quantum Matter, Beijing, China

**Keywords:** Perovskite, Single-crystalline thin film, Hydrophobic, Photodetector, Gain

## Abstract

Modern electronic and photonic devices rely on single-crystalline thin film semiconductors for high performance and reproducibility. The emerging halide perovskites have extraordinary electronic and photonic properties and can be synthesized via low cost solution-based methods. They have been used in a variety of devices with performance approaching or over the devices based on conventional materials. However, their solution based growth method is intrinsically challenge to grow large scale single-crystalline thin film due to the random nucleation and isotropous growth of the crystal. Here, we report the growth of centimeter-scale perovskite single-crystalline thin films by controlling the nucleation density and growth rate of the crystal under a spatially confined growth condition. The hydrophobic treatment on substrates inhibits nucleation and accelerates the growth of single-crystalline thin film, providing enough space for initial nucleus growing up quickly without touching each other. Single-crystalline perovskite thin-film with an aspect ratio of 1000 (1 cm in side length, 10 μm in thickness) has been successfully grown. The low trap density and the high mobility of the as-grown thin film show a high crystallinity. The photodetector based on the perovskite thin film has achieved a gain ~ 10^4^, benefitting from the short transit time of the carries due to the high mobility and thin thickness of the active layer. Our work opens up a new route to grow large scale perovskite single-crystalline thin films, providing a platform to develop high- performance devices.

## Introduction

Single-crystalline thin film (SC-TF) materials provide state-of-the-art performance in modern electronic and photonic devices [1–6]. The growth of conventional SC-TFs requires high vacuum and high temperature conditions. Recently, halide perovskites that can be grown by solution-based methods are emerging as a new generation of semiconductor materials with high device performance. To date, they have been used in various directions including solar cells [7–18], photodetectors [19–28], light emitting diodes and lasers [29–34], laser cooling devices [35, 36], and high-energy radiation detection [37–39].

However, solution-based growth methods are intrinsically challenged when it comes to growth of macroscale SC-TF, owing to the random nucleation and isotropous growth of the crystal. To date, large area high performance perovskite devices are based on polycrystalline thin films [11–19, 22–28]. On the other hand, liquid growth strategies including cooling crystallization [2, 40, 41], anti-solvent diffusion assisted crystallization [42, 43], and inverse temperature crystallization [28, 44, 45] have been developed to grow macroscale single-crystalline halide perovskites. However, the aspect ratios of these macroscale single crystals had been about unity depending on the isotropous growth rate in solution. Therefore, methods based on space-confine [4, 46–50], mechanical cutting [48], surface tension [51] and microjetting [52] have been introduced to grow perovskite crystals with large scale. However, the SC-TFs made by these methods are with limited aspect ratio. For that grown in a thin gap, random nucleation results in a high nucleation density, which limits the size of single-crystalline flakes. For that grown in a thick gap, or made by mechanical cutting technique, the films can be large but with thick thickness. Hydrophobic treatment of the substrate has been employed to get a larger aspect ratio [6, 16, 53, 54], however, the reported films are limited to millimeter-scale, and the effects of hydrophobic surface on nucleation density and growth rate have remained unexplored.

Here, we report the growth of centimeter-scale MAPbBr_3_ single-crystalline thin films with thickness down to 10 μm level by substrate surface engineering and space-confined growth. We further reveal the effects of hydrophobic surface on nucleation density and growth rate by treating the substrates with different solvent contact angles. The aspect ratio (side length over thickness) of the single-crystalline thin film exceeds 1000. The trap density of the as-grown thin film is on the order of 10^11^ cm^−3^, and the mobility is more than 60 cm^2^/V s. These special properties make our material outstanding from the perovskite SC-TFs (Additional file 1: Table S1). We fabricate photodetector based on the grown single-crystalline perovskite thin film. The device shows a gain ~ 10^4^, benefitting from the short transit time of the carries due to the high mobility and thin thickness of the active layer.

## Results and discussion

**Fig. 1:**
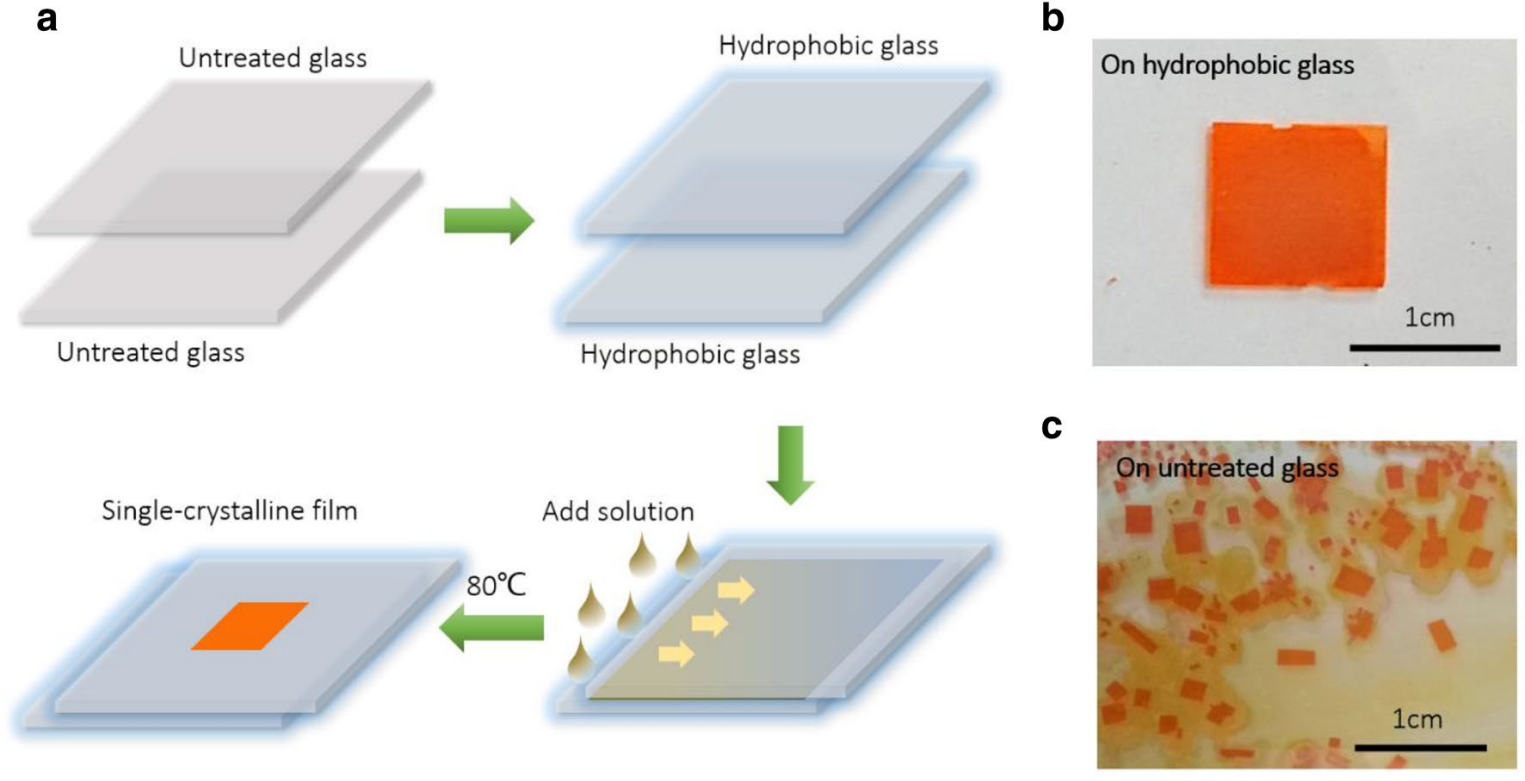
Growth of perovskite SCTF. **a** Schematic diagram of the growth process. The surfaces of two glass substrates are treated to be hydrophobic and put face-to-face to create a confined space. We then keep a constant growth temperature of 80 °C and add the precursor into the gap continuously via capillarity effect to grow the SCTFs. **b** Photograph of a centimeter-scale perovskite SCTF grown inside two hydrophobic substrates. **c** Millimeter-scale single-crystalline flakes grown inside two untreated substrates

Figure 1a shows the schematic diagram of the growth process. The surfaces of two glass substrates are treated to be hydrophobic and put face-to-face to create a confined space. We then keep a constant growth temperature of 80 °C and continuously add the precursor into the gap via capillarity effect, providing enough precursor solution for continuously growth of the film, which is indispensable to grow centimeter-scale SC-TFs. Hydrophobic treatment of the substrates is essential to grow the centimeter-scale perovskite SC-TFs, because it not only modifies the nucleation process to limit the number of crystal nucleus formed inside the gap, but also accelerates the growth rate of the film. Figure 1b shows the photograph of a centimeter-scale perovskite SC-TF grown inside two hydrophobic substrates. In contrast, Fig. 1c shows the photograph of millimeter-scale single-crystalline flakes grown inside two untreated substrates.

**Fig. 2:**
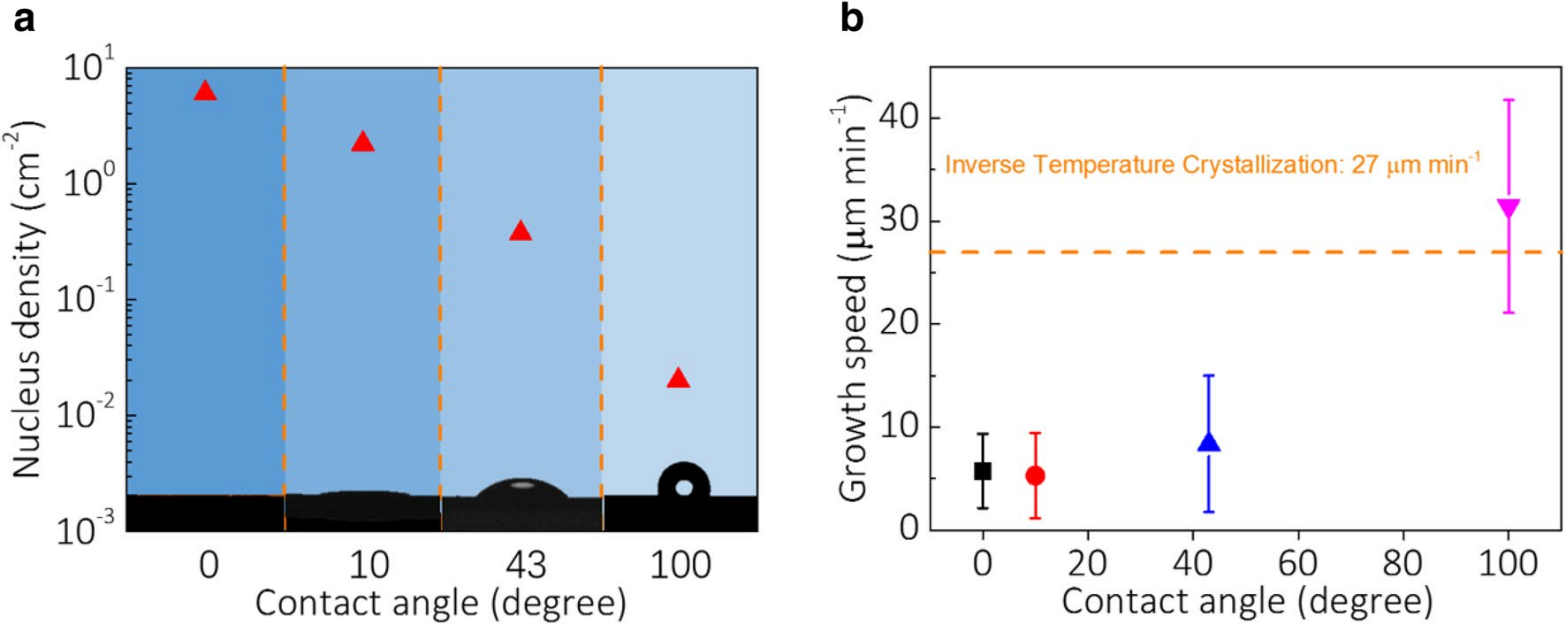
Influences on the SC-TF growth by surface engineering of substrates. **a** The measured nucleus densities on the substrates with different solvent contact angles resulted by different surface treatment conditions. **b** Growth rates of perovskite single-crystalline thin films on these substrates

Figure 2a shows the measured nucleus densities on the substrates with different solvent contact angles resulted from different surface treatment conditions (Additional file 1: Figure S1). As the contact angle of the substrate increases, the nucleation density of the substrate surface decreases by 300 times from completely hydrophilic to hydrophobic. Also, we have measured the growth rates of perovskite SC-TFs on these substrates, which are shown in Fig. 2b. The growth rate of the crystal increases with the increase of the contact angle. The average growth rate of the crystal in the gap of hydrophobic substrates is 31.5 μm min^−1^, which is 6 times of that on completely hydrophilic substrates. Notably, the growth rate in the micrometer scale gap of hydrophobic substrates is even higher than the growth rate of a single-crystalline bulk perovskite in completely free solution space (27.0 μm min^−1^) [28].

The nucleation mechanisms on hydrophilic and hydrophobic substrates are shown in Additional file 1: Figure S2a, b, respectively. The attraction between surface atoms with solvent molecules and solvated precursor ions on hydrophilic surface is comparably stronger than hydrophobic surface [6, 55]. Strong attraction of surface to the pre-existing clusters will fix more clusters on the substrate surface and block the re-dissolution of ions from pre-existing cluster surfaces. So, the existing cluster becomes more stable and easier to grow into crystal nucleus. In addition, the reaction heat released during cluster growth can be dissipated more rapidly through the surfaces due to a relatively higher interaction intensity, which is beneficial to the formation of nucleus. As a result, the nucleation free energy barrier on hydrophilic surface is relatively lower than that on a hydrophobic condition, yielding a comparably higher nucleation rate [56, 57]. Meanwhile, the precursor ions and solvent that get close to the surface will be attracted and then captured by the hydrophilic surface, which will slow the diffusion of ions, leading to a slower crystal growth rate [14, 58–60]. Therefore, hydrophobic modification on substrates reduces nucleation density and accelerates the growth rate of SC-TFs.

**Fig. 3:**
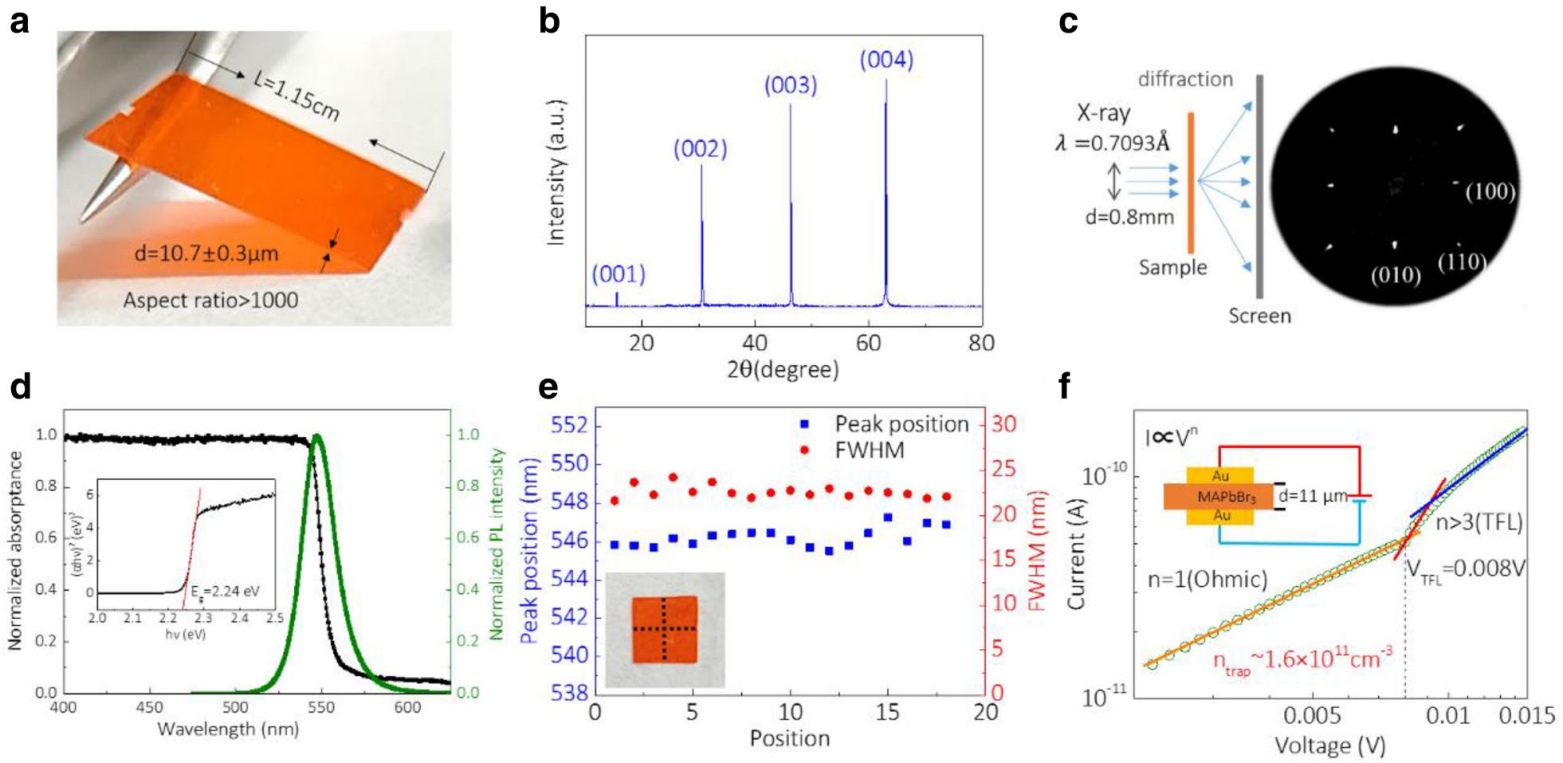
Characterization of perovskite SC-TFs. **a** The grown MAPbBr3 SC-TF peeled off from the substrates. The thickness of the SC-TF is 10.7 ± 0.3 μm and the length is 1.15 cm, giving an aspect ratio of 1074. **b** X-ray diffraction spectrum of the MAPbBr_3_ SC-TF, which shows that the thin film is single crystal in cubic phase and the crystal orientation of the film in vertical direction is (001). **c** The schematic and Bragg spots for transmission X-ray microscopy measurement. **d** Absorption spectrum and steady-state PL spectrum of the prepared MAPbBr_3_ SC-TF. Insets: absorptance versus energy plot of the film, determining the optical bandgap as 2.24 eV. **e** PL spectra at different positions which formed the cross line on the same film (inset). The almost unchanged peak position and full width half maximum show highly uniformity of the optical property due to the uniform crystallization of the film. **f** The dark I-V curve used for the trap-state-density measurement

Figure 3a shows the photograph of the grown MAPbBr_3_ SC-TF peeled off from substrates. The thickness of the SC-TF is 10.7 ± 0.3 μm and the length is 1.15 cm, giving an aspect ratio of 1074 (Additional file 1: Figure. S3). X-ray diffraction (XRD) characterization of the SC-TF (Fig. 3b) shows that the thin film is single crystalline with cubic phase and the crystal orientation of the film in vertical direction is (001). The full width half maximum (FWHM) of (002) peak in X-ray diffraction spectrum is 0.035^o^ (Additional file 1: Figure. S4), confirming the high crystallinity of the SC-TF. The transmission X-ray microscopy results are shown in Fig. 3c. The scattered diffraction spots rather than a continuous arc prove the single-crystalline character of the grown material. And the crystal orientation in horizontal direction along the sides of the square shape are (100) and (010).

Figure 3d shows the absorption spectrum and steady-state photoluminescence (PL) spectrum of the prepared MAPbBr_3_ SC-TF. The band gap of the film is calculated as 2.24 eV and the steady-state PL spectrum shows the emission peak located at 546 nm with a FWHM of 22 nm. In order to characterize the optical uniformity of the film, we measured PL spectra at different positions which formed the cross line on the same film. As shown in Fig. 3e, the peak positions are located at 546.2 $\pm 0.5$ nm and the FWHM of the PL spectra is 22.6 $\pm 0.7$ nm. The almost unchanged peak position and FWHM show highly uniformity of the optical property due to the uniform crystallization of the film.

The trap density of the film is measured with the space-charge-limited current analysis under different biases to characterize the crystallinity of MAPbBr_3_ SC-TF quantitatively. For the electrical characterization, Au electrodes are thermally deposited on both top and bottom surfaces of the free-standing film. As shown in Fig. 3f, the I-V characteristics of the film changes at V_TFL_ = 0.008 V. Below 0.008 V, the I-V curve shows an Ohmic contact characteristic. Above 0.008 V, the current rises sharply with voltage, resulted from all trap states being filled by injected charge carriers. The trap density is gotten from $n_{trap} = \frac{{2\varepsilon_{r} \varepsilon_{0} V_{TFL} }}{{ed^{2} }} = 1.6 \times 10^{11} {\text{cm}}^{ - 3}$, where =11 μm is the thickness of the film, $\varepsilon_{r} = 22.5$ is the relative dielectric constant, and $\varepsilon_{0}$ is the vacuum permittivity. The trap density of MAPbBr_3_ SC-TF is 6 orders of magnitude lower than polycrystalline films fabricated by spin-coating methods [28]. We also characterize the mobility of our large SC-TF via Hall effect which is over 60 cm^2^/V s (Additional file 1: Figure. S5), orders of magnitude higher than MAPbBr_3_ polycrystalline films fabricated by spin-coating methods [48].

**Fig. 4:**
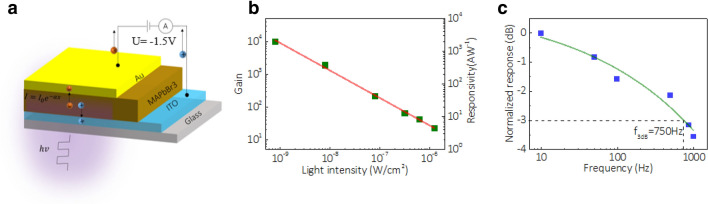
Characterization of MAPbBr_3_ SC-TF photodetector. **a** Structure of the device. The film is sandwiched in the ITO substrate and top Au electrode, forming a metal–semiconductor-metal structure. **b** Gain and responsivity of the device at different incident power density. The largest gain is ~ $10^{4}$ which benefits from the high crystallinity and thin thickness of the active layer. **c** 3 dB bandwidth of the device is measured as 750 Hz

For photonic devices, the high crystallinity and thin thickness of the film will reduce the recombination probability of photo-generated carriers, leading to a more efficient photon-to-carrier conversion and hence to a high performance of photodetector and solar cells [61]. Here, we fabricate the photodetector based on the grown SC-TF, as shown in Fig. 4a. The film is grown on the ITO glass which is employed as one of the electrodes. Another Au electrode is fabricated on the top of the film, forming a metal–semiconductor-metal structure (Additional file 1: Figure. S7a, b). The I-V curves under light and dark conditions are shown in Additional file 1: Figure. S7c, which clearly show rectifying behavior as the ITO electrode is grounded, indicating that a Schottky barrier is formed at MAPbBr_3_/Au interface [54]. We measured gain of the device at -1.5 V, where the device shows a pronounced response. Gain of the device here is from the difference between the carriers lifetime in trap state and the carriers transit time. It can be calculated by $\frac{{{{\left( {J_{light} - J_{dark} } \right)} \mathord{\left/ {\vphantom {{\left( {J_{light} - J_{dark} } \right)} e}} \right. \kern-0pt} e}}}{{{P \mathord{\left/ {\vphantom {P {hv}}} \right. \kern-0pt} {hv}}}}$, where *e* is electronic charge, $hv$ is the incident photon energy, *P* is the incident power density, $J_{light}$ and $J_{dark}$ are the current density under light and dark condition [61, 62]. Figure 4b shows illumination power dependence of gain and corresponding responsivity $R = \frac{G}{{{{hv} \mathord{\left/ {\vphantom {{hv} e}} \right. \kern-0pt} e}}}$ of our device at − 1.5 V. Gain continuously increases with the decrease of the incident power and reaches the maximum of ~ $10^{4}$ at 8 nW cm^−2^. The trend of the illumination power dependent gain comes from the saturation of the trap states by the large amount of the external injected electrons [54]. With the decrease of the incident power, the ratio of the trapped electrons increases, resulting in an increase in the gain. We also characterized the transient photocurrent response of the photodetector at 0 V, as shown in Additional file 1: Figure S7d. The rise time and the fall time are 170 µs and 320 µs, respectively. The 3 dB bandwidth of the photodetector is measured as 750 Hz, shown in Fig. 4c. The high gain and fast response of the device benefit from the short transit time of the carries, which origins from the high mobility and thin thickness of the single crystalline thin film active layer.

## Conclusions

In summary, we have proposed an approach to produce large-scale single-crystalline perovskite thin films via surface engineering under spatially confined growth condition. Hydrophobic treatment on the substrate can reduce nucleation density and accelerate the growth rate of SC-TFs. The as-grown perovskite SC-TFs has 1 cm side length with a 10 μm thickness. The large SC-TFs produced also show very good uniformity in crystallinity quality. The trap density of the as-grown thin film is on the order of 10^11^ cm^−3^, and the mobility is over 60 cm^2^/V s. The photodetector based on the perovskite thin film has achieved a gain ~ 10^4^, benefitting from the short transit time of the carries due to the high mobility and thin thickness of the active layer. Our work opens a new and alternative facsimile route to grow large scale SC-TFs. These results might develop further exploration of on-chip fabrication of large-scale high performance devices with hybrid perovskite SC-TFs.

## Methods/experimental

### Materials

Dimethyldimethoxysilane (> 95%) was obtained from Heowns. Hydrobromic acid (> 40.0wt. %), methylamine alcohol solution (30.0wt. %), acetone (> 99.7%), ethanol (> 99.7%), isopropanol (> 99.7%), dichloromethane (> 99.0%), sulfuric acid (95.9wt %) were all obtained from Sinopharm Chemical Reagent Co., Ltd (SCRC). Dimethylformamide (> 99.9%) were purchased from Concord Technology (Tianjin) Co., Ltd. Lead(II) bromide (99%), were purchased from Aladdin reagent. Deionized water with a resistivity of 18.2 MΩ cm (Milli-Q) was used for all rinsing processes. All reagent used without further purification. ITO-coated glass substrates (8–12 Ωsq^−1^) were purchased from BOE Technology Group Co., Ltd. Glass microscope slides (100.0 × 100.0 mm) were purchased from Haimen experimental equipment factory (Jiangsu).

### Synthesis of perovskite precursor

MABr (MA = CH_3_NH_3_^+^) was synthesized through the reaction of methylamine with hydrobromic acid. An equimolar amount of hydrobromic acid (> 40.0wt. % in water) was added dropwise into the methylamine (30.0wt. % in methanol) at ice bath under stirring state. Then the mixture solution was stirred for 2 h. Removal of the solvent was followed by recrystallization from ethanol to yield MABr crystals, and finally dried in a vacuum oven at 60 °C overnight.

### Preparation of hydrophobic reaction solution

40 ml of isopropanol, 3.5 ml of dimethyldimethoxysilane and 160  ul of sulfuric acid were added to a 50 ml plastic centrifuge tube. The solution was swirled to fully mix and place for 30 min at room temperature before use.

### Process of surface treatment

Hydrophobic treatment: We cleaned the glass slide use acetone, ethanol and deionized water in turn in an ultrasonic cleaning machine, then let the glass substrate dry naturally. The contact angle of the cleaned glass slide is 43°. Then we submerged the cleaned glass in the reactive solution for 60 s and withdrawned gradually. The substrate was allowed to dry about 5 min at room temperature (25 °C). Ethanol and water were used to rinse the glass surface. The contact angle of the hydrophobic substrate is 100^o^.

Hydrophilic treatment: We use oxygen plasma treatment (PDC-32G Harrick Plasma Cleaner) for the hydrophilic treatment of the glass substrate. Glass substrates were treated with low power oxygen plasma for 3 min, the contact angle is 10°. Glass substrates were treated with low power oxygen plasma for 15 min, and the contact angle is almost 0^o^.

### Growth of perovskite single-crystalline thin films

To grow single-crystalline halide perovskite thin films, we introduced anisotropic growth environment into the growth. First, PbBr_2_ and MABr (1/1 by molar, 1 M) were dissolved in *N*,*N*-dimethylformamide (DMF). Then a piece of glass slice was put on the top of another glass slice to form the thin gap. Then the perovskite precursor solution was added to the edge of the substrate, and it was absorbed into the gap by capillarity. Next, this system was put in 80 °C environment to grow the film by inverse temperature crystallization. Until the growth of single-crystalline thin films were completed, then the perovskite precursor is added. Finally, the crystals continue to grow until they reach a size of 1 cm after several cycles of precursor addition.

### Single-crystalline thin film characterization

The thickness of the film was measured by a The Dektak^®^ 150 stylus surface profiler. The XRD measurement was applied to the film by PANalytical X’Pert Pro with a Cu-Ka X-ray (λ = 1.541874 Å) target, V = 40 kV, I = 40 mA. The cross section analysis was carried out using scanning electron microscope (Helios Nanolab 600i). The absorption spectra were captured with PerkinElmer, Lambda 950. PL spectrum excited by a 405 nm laser was measured by a confocal Raman microscopic system (Horiba, Labram HR800). The Hall effect was characterized measured with a Nanometrics HL5500 Hall system. I–V measurement was done using a Keithley 4200-SCS. All above measurements were carried out at room temperature.

### Preparation and characterization of the photodetector

We fabricate MAPbBr_3_ SC-TF photodetector based on a MAPbBr_3_/Au Schottky diode. MAPbBr_3_ SC-TF was first grown on the ITO substrate. Following that a hard mask had been put on the top surface of the film. A square Au electrode (50 nm in thickness, 2 mm × 2 mm in area) was then thermally deposited on the top surface of the film in the vacuum chamber.

As shown in Additional file 1: Figure S6. A device is fixed on the probe station under a microscope, and is electrically connected to the electrical characterization equipment. A source meter (Keithley 4200) is used to bias the device and measure the corresponding current. In light response measurement, a 2 MΩ resistance is connected in series with the detector, and the voltage signal is measured by an oscilloscope. An optical chopper is placed between the lens L1 and L2 to modulate the continuous-wave laser beam at 405 nm. The intensity of the laser beam is adjusted by an optical attenuator and the power is measured by a power meter.

## Supplementary information

**Additional file 1: Figure S1.** Nucleation density on different substrates. **Figure S2.** Schematic illustrations of the nucleus mechanism. **Figure S3.** Aspect ratio of MAPbBr_3_ SC-TF. **Figure S4.** Crystallinity of MAPbBr_3_ SC-TF. **Figure S5.** Mobility of MAPbBr_3_ SC-TF. **Figure S6.** Device measurement setup. **Figure S7.** Characterization of the photodetector made of MAPbBr_3_ SC-TF. **Table S1.** Comparison of the properties of the perovskite SC-TF materials.

## Data Availability

All data that support the plots within this paper and other findings of this study are available from the corresponding author on reasonable request.

## Abbreviations

SC-TF: Single crystal thin film
MA: Methylamine
MABr: Bromide methylamine
PbBr_2_: Lead bromide
PL: Photoluminescence
XRD: X-ray diffraction
FWHM: Full width half maximum
ITO: Indium Tin Oxide
DMF: *N*,*N*-dimethylformamide
