# Supplementary material for: Growth of centimeter-scale perovskite single-crystalline thin film via surface engineering
Source: Nano Converg. 2020 Jul 20;7:25. doi: 10.1186/s40580-020-00236-5 (PMC7371768; doi:10.1186/s40580-020-00236-5)
Supplement: Supplementary file 1 — Additional file 1: Figure S1. Nucleation density on different substrates. Figure S2. Schematic illustrations of the nucleus mechanism. Figure S3. Aspect ratio of MAPbBr3 SC-TF. Figure S4. Crystallinity of MAPbBr3 SC-TF. Figure S5. Mobility of MAPbBr3 SC-TF. Figure S6. Device measurement setup. Figure S7. Characterization of the photodetector made of MAPbBr3 SC-TF. Table S1. Comparison of the properties of the perovskite SC-TF materials. [file 40580_2020_236_MOESM1_ESM.pdf]

Additional file

Growth of centimeter-scale perovskite single-crystalline thin film via surface engineering

Yu-Hao Deng[1,2], Zhen-Qian Yang[1,2], Ren-Min Ma[1,2*]

[1] State Key Lab for Mesoscopic Physics and School of Physics, Peking University, Beijing, China

[2] Frontiers Science Center for Nano-optoelectronics & Collaborative Innovation Center of Quantum Matter, Beijing, China

* Correspondence should be addressed to renminma@pku.edu.cn

Contents

1. Growth of $MAPbBr_3$ SC-TF 2
2. Nucleation density on different substrates. 3
3. Schematic illustrations of the nucleus mechanism 4
5. Crystallinity of $MAPbBr_3$ SC-TF. 6
6. Mobility of $MAPbBr_3$ SC-TF. 7
7. Device measurement setup 8
8. Characterization of the photodetector made of $MAPbBr_3$ SC-TF. 9
9. Comparison of the properties of the perovskite SC-TF materials. 10

References 11

1. Growth of $MAPbBr_3$ SC-TF

To grow single-crystalline halide perovskite thin films, we introduced anisotropic growth environment into the growth. First, $PbBr_2$ and MABr (1/1 by molar, 1 M) were dissolved in N, N-dimethylformamide (DMF). Then a piece of glass slice was put on the top of another glass slice to form the thin gap. Then the perovskite precursor solution was added to the edge of the substrate, and it was absorbed into the gap by capillarity. Next, this system was put in 80 ºC environment to grow the film by inverse temperature crystallization. Until the growth of single-crystalline thin films were completed, then the perovskite precursor is added. Finally, the crystals continue to grow until they reach a size of 1cm after several cycles of precursor addition.

In order to control the nucleation density for large area film growth without boundaries, the glass slices were hydrophobic molecules before used to form high nucleation energy barrier. Hydrophobic treatment to glass surface can increase the nucleation energy barrier, leading to an appropriate condition for large area film growth. [1] The hydrophobic process is based on the procedure described by Wang L et al. [2] 40 ml of isopropanol, 3.5 ml of dimethyldimethoxysilane (DMDMS) and 160 ul of sulfuric acid were added to a 50 ml plastic centrifuge tube. The solution was swirled to fully mix and place for 30 min at room temperature before use. We cleaned the glass slides with a side length of 10 cm use acetone, ethanol and deionized water in turn in an ultrasonic cleaning machine, then submerged the glasses in the reactive solution for 60 seconds and lifted the glasses out gradually. The substrate was allowed to dry about 5 min at room temperature (25 °C). Ethanol and water were used to rinse the glasses surface.

2. Nucleation density on different substrates.

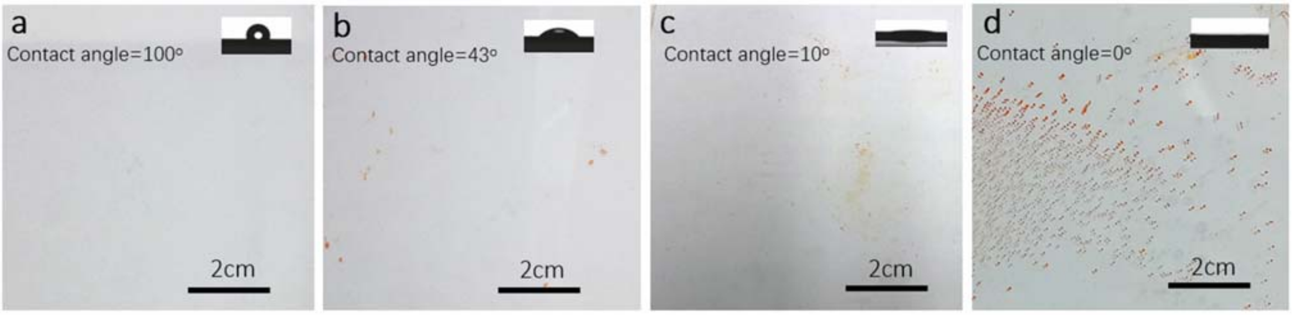

Figure S1. Photographs of nucleation on the substrates with different contact angle. (a) Hydrophobically treated substrates. (b) Untreated glass slides. (c-d) Hydrophilic surfaces. Glass slides were treated with oxygen plasma treatment for 2 min (b) and 15 min (d) to induce hydrophilic surfaces. The number of nucleus can be seen in the pictures and counted.

3. Schematic illustrations of the nucleus mechanism

The interaction and random collision of ions in the solution will form clusters, which are accompanied by continuous re-dissolution and generation. For hydrophilic surface (high energy), the attraction of surface atoms to precursor ions is comparably stronger than hydrophobic surface (low energy). The attractions affect the nucleation free energy barrier and crystal growth rate.

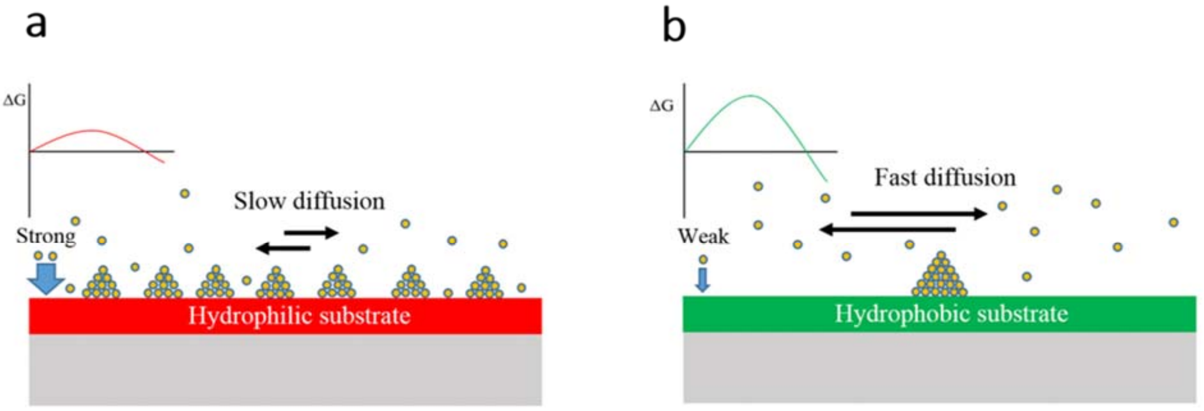

Figure S2. Schematic illustrations of the nucleus mechanism on hydrophilic (a) and hydrophobic (b) substrates. Inset: the energy barrier of forming nucleus.

4. Aspect ratio of $MAPbBr_3$ SC-TF

The scanning curve of the step profiler is shown in Figure. S3. The thickness of $MAPbBr_3$ SC-TF is 10.7 ± 0.3 μm and length is 1.15 cm, corresponding to an aspect ratio of 1074.

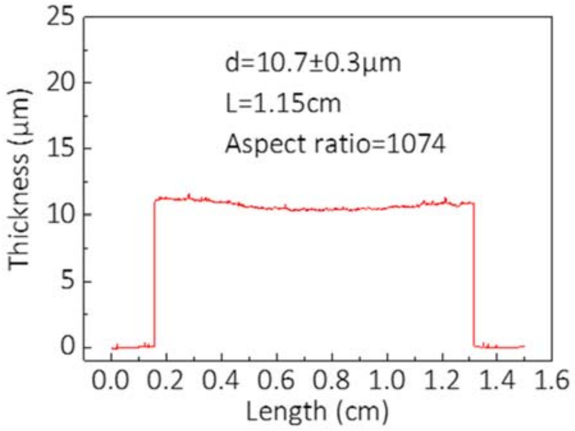

Figure S3. Scanning curve of the step profiler.

5. Crystallinity of $MAPbBr_3$ SC-TF.

Figure S4 shows the detail information of (002) peak, the data were fitted by Lorentzian formula. And the FWHM is 0.035°, indicating the excellent crystallinity of the film. As a qualitative indication of crystallinity, this value is better than that of reported bulk $MAPbBr_3$ crystal.[3] The split of the peak is because of the adjacent wavelength of Kα1 (1.54056 Å) and Kα2 (1.54439 Å) with 1:2 intensity in the Cu Kα X-ray source.

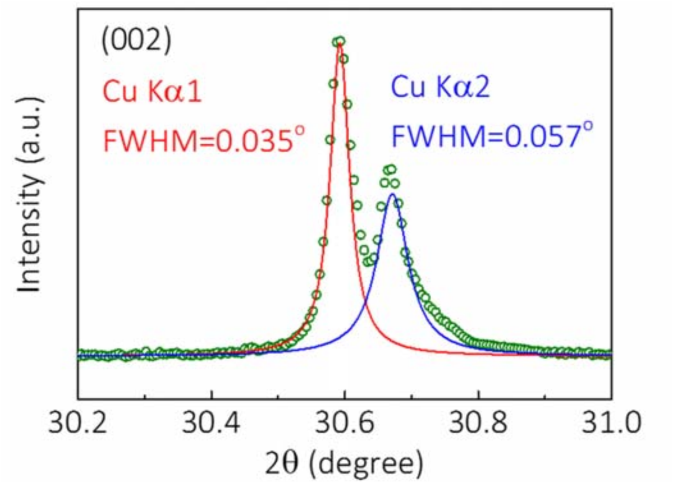

Figure S4. Detail information of (002) peak.

6. Mobility of $MAPbBr_3$ SC-TF.

The schematic diagram of Hall effect measurement is shown as **Figure S5a**. Four symmetrical Au electrodes were thermally evaporated onto the surface of free standing single-crystalline thin film, as shown in **Figure S5b**. The thickness of the electrodes is 100 nm. The Hall effect measurement result shows the mobility of the single-crystalline film is over 60 cm^2/Vs, which is consistent with the bulk material. The mobility is about 200 times higher than the traditional spin-coating film due to the lower trap density and excellent crystallinity.[4] Also, the Hall effect shows that the film is p-type doping.

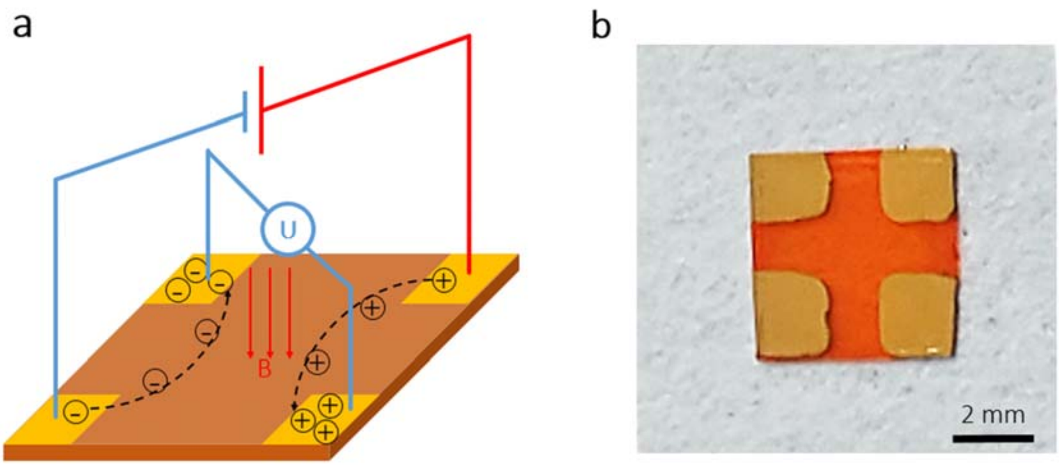

Figure S5. The schematic diagram and the photograph of the device for Hall effect measurement.

7. Device measurement setup

The measurement setup is shown in **Figure S6**. A device is fixed on the probe station under a microscope, and is electrically connected to the electrical characterization equipment. A source meter (Keithley 4200) is used to bias the device and measure the corresponding current. In light response measurement, a 2 MΩ resistance is connected in series with the detector, and the voltage signal is measured by an oscilloscope. An optical chopper is placed between the lens L1 and L2 to modulate the continuous-wave laser beam at 405 nm. The intensity of the laser beam is adjusted by an optical attenuator and the power is measured by a power meter.

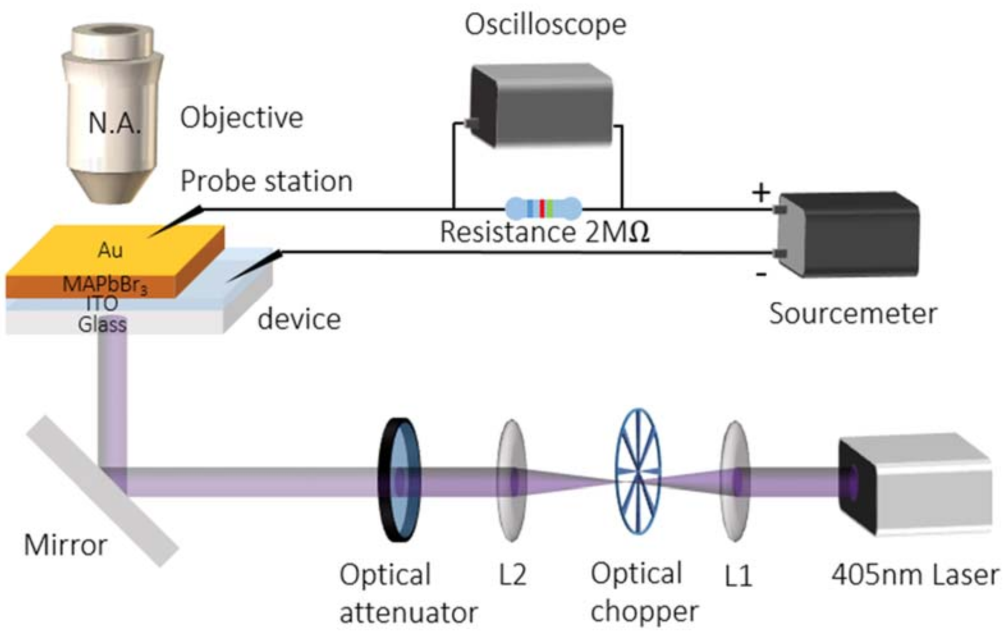

Figure S6. Schematic of the measurement setup.

8. Characterization of the photodetector made of $MAPbBr_3$ SC-TF.

Figure S7a shows photograph of the photodetector. We fabricate $MAPbBr_3$ SC-TF photodetector based on a $MAPbBr_3$/Au Schottky diode. $MAPbBr_3$ SC-TF was first grown on the ITO substrate. Following that a hard mask had been put on the top surface of the film. A square Au electrode (50 nm in thickness, 2 mm × 2 mm in area) was then thermally deposited on the top surface of the film in the vacuum chamber. **Figure S7b** shows the cross-profile SEM image of a typical device. **Figure S7c** shows the I-V curves of the device under dark condition and an illumination condition of 800 nW cm^{-2}. Under both conditions, I-V curves clearly show rectifying behavior. The diode is open when negative bias is applied, indicating that a Schottky barrier is formed at MAPbBr3/Au interface. The Time domain response of the device at 0 V is shown in **Figure S7d**. The rise and fall times are 170 μs and 320 μs respectively.

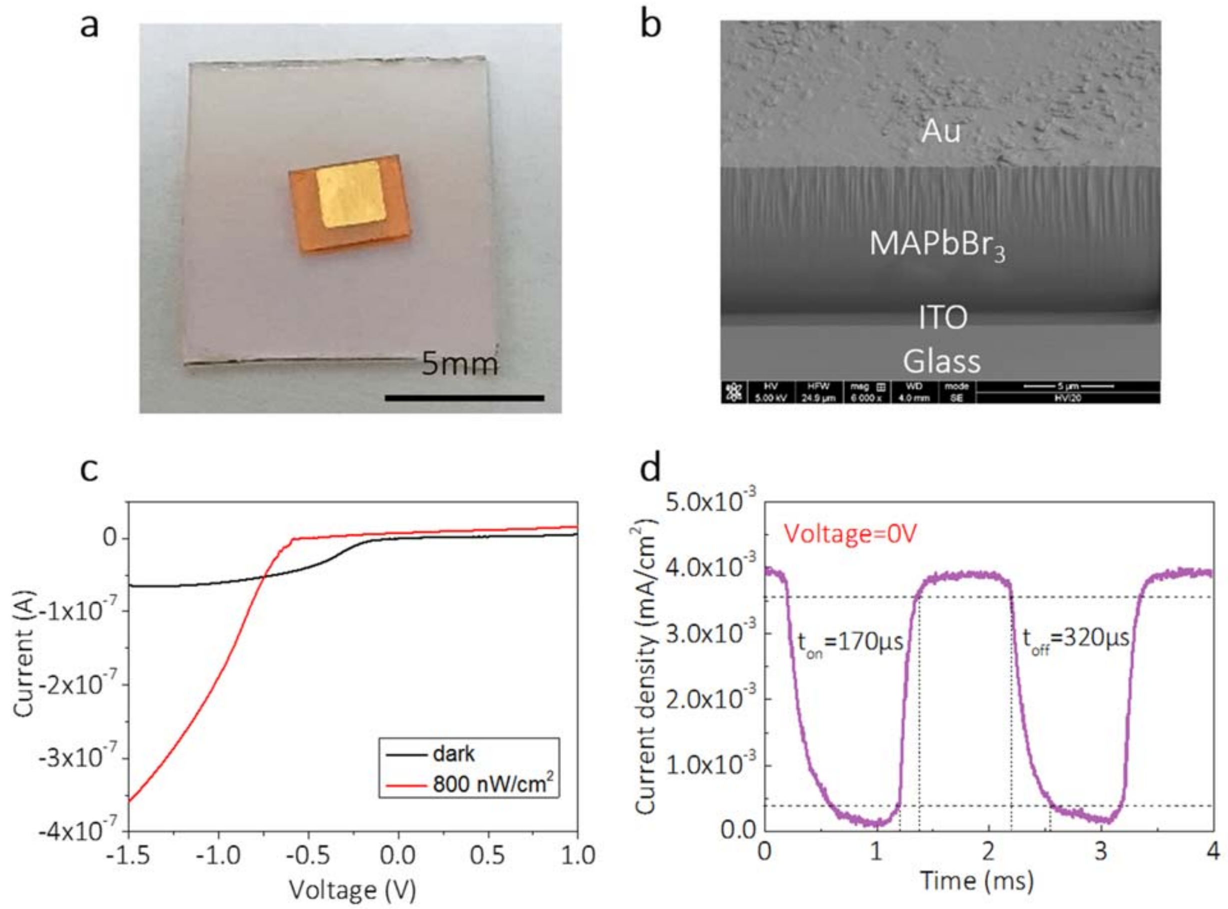

Figure S7. Characterization of the photodetector made of the $MAPbBr_3$ SC-TF. (a) Optical photograph of the device. (b)The cross-profile SEM image of the photodetector. The single-crystalline film shows no grain boundaries. (c) The I-V curves under light and dark condition. (d) The transient photocurrent response of the photodetector.

9. Comparison of the properties of the perovskite SC-TF materials.

Table S1. Comparison of the properties of the perovskite SC-TF materials.

Size	Year	Material	Length	Thickness	Aspect ratio	Trap density (cm^{-3})	Mobility ($cm^2/V{\cdot}s$)	Ref
~Centimeter-scale	2020	$MAPbBr_3$	1.15 cm	10.7 μm	1074	1.6×10^{11}	60	Our results
	2019	$MAPbI_3$	1 cm	170 μm	58.8	2.4×10^{10}	/	5
	2017	$MAPbBr_3$	0.6 cm	16 μm	375	2.5×10^{10}	23.7	6
	2016	$FAPbI_3$	2 cm	100 μm	200	1.3×10^{10}	40	7
	2016	$MAPbI_3$	0.56 cm	150 μm	37.5	6.0×10^{8}	39.6	8
~Millimeter-/ submillimeter -scale	2020	$MAPbBr_3$	0.35 mm	8 nm	44000	/	/	9
	2018	$CsPbBr_3$	0.69 mm	500 nm	1380	1.8×10^{10}	1770	10
	2018	$MAPbBr_3$	0.67 mm	420 nm	1600	$\sim10^{11}$	59	11
	2017	$MAPbBr_3$	3 mm	10 μm	300	5.3×10^{11}	/	12
	2016	$MAPbBr_3$	2.2 mm	~2.9 μm	~750	$\sim10^{11}$	/	13
	2016	$MAPbBr_3$	~1 mm	~10 nm	$\sim10^{5}$	4.8×10^{10}	15.7	14

References

[1] T. Suni, K. Henttinen, I. Suni, J. Mäkinen, *J. Electrochem. Soc.*, 2002, *149*, G348-G351.

[2] L. Wang, T.J. McCarthy, *Angew. Chem. Int. Edi.*, 2016, 55, 244-248.

[3] W. Peng, L. Wang, B. Murali, K. T. Ho, A. Bera, N. Cho, C. F. Kang, V. M. Burlakov, J. Pan, L. Sinatra, C. Ma, W. Xu, D. Shi, E. Alarousu, A. Goriely, J. He, O. F. Mohammed, T. Wu, O. M. Bakr, *Adv. Mater.*, 2016, *28*, 3383-3390.

[4] M. I. Saidaminov, V. Adinolfi, R. Comin, A. L. Abdelhady, W. Peng, I. Dursun, M. Yuan, S. Hoogland, E. H. Sargent, O. M. Bakr, *Nat. Commun.*, 2015, *6*, 8724.

[5] J. Gao, Q. Liang, G. Li, T. Ji, Y. Liu, M. Fan, Y. Hao, S. Liu (Frank), Y. Wu, Y. Cui, *J. Mater. Chem. C*, 2019, 7, 8357-8363.

[6] H. Rao, B. Chen, X. Wang, D. Kuang, C. Su, *Chem. Commun.*, 2017, 53, 5163-5166.

[7] Y. Liu, J. Sun, Z. Yang, D. Yang, X. Ren, H. Xu, Z. Yang and S. Liu, *Adv. Opt. Mater.*, 2016, 4, 1829-1837.

[8] Y. Liu, Y. Zhang, Z. Yang, D. Yang, X. Ren, L. Pang and S. Liu, *Adv. Mater.*, 2016, 28, 9204-9209.

[9] Y. Bai, H. Zhang, M. Zhang, D. Wang, H. Zeng, J. Zhao, H. Xue, G. Wu, J. Su, Y. Xie, *Nanoscale*, 2020, 12, 1100-1108.

[10] Z. Yang, Q. Xu, X. Wang, J. Lu, H. Wang, F. Li, L. Zhang, G. Hu, C. Pan, *Adv. Mater.*, 2018, 30, 1802110.

[11] Z. Yang, Y. Deng, X. Zhang, S. Wang, H. Chen, S. Yang, J. Khurgin, N. Fang, X. Zhang, R. Ma, *Adv. Mater.*, 2018, 30, 1704333.

[12] A. Zhumekenov, V. Burlakov, M. Saidaminov, A. Alofi, M. Hague, B. Turedi, B. Davaasuren, I. Dursun, N. Cho, A. El-Zohry, et al., *ACS Energy Lett.*, 2017, 2, 1782-1788.

[13] W. Peng, L. Wang, B. Murali, K. Ho, A. Bera, N. Cho, C. Kang, V. Burlakov, J. Pan, L. Sinatra, et al., *Adv. Mater.*, 2016, 28, 3383-3390.

[14] Y. Chen, Q. Ge, Y. Shi, J. Liu, D. Xue, J. Ma, J. Ding, H. Yan, J. Hu, L. Wan, *J. Am. Chem. Soc.*, 2016, *138*, 16196-16199.
